# Clinical outcome after thrombectomy in patients with MeVO stroke: importance of clinical and technical factors

**DOI:** 10.1007/s00415-023-12025-1

**Published:** 2023-10-17

**Authors:** Terje Nome, Brian Enriquez, Cecilie G. Nome, Bjørn Tennøe, Christian G. Lund, Mona Skjelland, Anne Hege Aamodt, Mona Beyer

**Affiliations:** 1 Division of Radiology and Nuclear Medicine, Oslo University Hospital, Oslo, Norway; 2 Department of Neurology, Oslo University Hospital, Oslo, Norway; 3 Institute of Clinical Medicine, University of Oslo, Oslo, Norway; 4 Division of Anatomy, Department of Molecular Medicine, Institute of Basic Medical Sciences, GliaLab and Letten Centre, University of Oslo, Oslo, Norway; 5 Department of Neuromedicine and Movement Science, The Norwegian University of Science and Technology, Trondheim, Norway

**Keywords:** Stroke, Medium vessel occlusion, Thrombectomy, Endovascular therapy, MRI

## Abstract

**Background and aims:**

Whereas high-level evidence has been proven for safety and efficacy of endovascular treatment (EVT) in large vessel occlusion (LVO) stroke, the evidence for EVT in medium vessel occlusion (MeVO) in both sexes and different age groupremains to be answered. The aim of this study was to evaluate the importance of clinical and technical parameters, focusing on sex, age and EVT procedural factors, on functional outcome in primary MeVO (pMeVO) strokes.

**Methods:**

144 patients with pMeVO in the MCA territory from the Oslo Acute Reperfusion Stroke Study (OSCAR) were included. Clinical and radiological data were collected including 90-day mRS follow-up.

**Results:**

Successful reperfusion with modified thrombolysis in cerebral infarction (mTICI) ≥ 2b was achieved in 123 patients (84%). Good functional outcome (mRS ≤ 2) at 90-day follow-up was achieved in 84 patients (61.8%). Two or more passes with stent retriever was associated with increased risk of SAH, poor mTICI and poor functional outcome. In average, women had 62 min longer ictus to recanalization time compared to men. Age over 80 years was significantly associated with poor outcome and death.

**Conclusion:**

In pMeVO patients, TICI score and number of passes with stent retriever were the main technical factors predicting mRS ≤ 2. Good clinical outcome occurred almost twice as often in patients under 80 years of age compared to patients over 80 years. Women with MeVO strokes had significant longer time from ictus to recanalization; however, this did not affect the clinical outcome.

## Introduction

Whereas high-level evidence has been proven for safety and efficacy of endovascular treatment (EVT) in large-vessel occlusion (LVO) stroke [1, 2], the evidence for EVT in medium-vessel occlusion (MeVO) remains to be answered. However, EVT is increasingly being performed in MeVO stroke and more studies are looking into the effect and outcome in these patients [3–7]. MeVOs are defined as occlusions of the M2/3 middle cerebral artery (MCA), A2/3 anterior cerebral artery and P2/3 posterior cerebral artery segments [3]. This type of stroke is estimated to account for 25–40% of all acute ischemic stroke cases with visible arterial occlusion [3]. The subgroup of patients with proximal M2 occlusions included in the LVO trials in the HERMES Collaboration benefited from EVT [8].

Based on their underlying mechanism, MeVO stroke can be classified as either primary (pMeVOs) or secondary (sMeVOs). Whereas pMeVOs occurs de novo, with etiology similar to LVOs, sMeVOs are due to clot migration or fragmentation of a LVO [9]. Thrombectomy in MeVOs carries a risk for embolization to new territories if the thrombus is dislocated or fragmented during the procedure. It is reasonable to assume that the risk of embolization to new vessel territories is greater with EVT for distal thrombi, as several vessel branches are passed in retrieving the thrombus. Distal cerebral arteries entail challenges related to navigation due to tortuosity, vessel diameter and longer distance from the puncture site. These factors implicate an increased risk of dissection, perforation, and vasospasm related to thrombectomy with stent retriever [10]. There is a need to explore whether the complexity and, potentially the increased risk of complications associated with EVT in MeVO patients, compared to patients with LVO, should have consequences for treatment indication and strategy [11].

The aim of this study was to evaluate functional outcome 90 days after EVT in patients with pMeVO in MCA territory with a focus on technical parameters such as hemorrhagic complications and embolization to new vessel territories. We also aimed to investigate the significance of clinical factors such as sex and age, which may aid in the selection of pMeVO patients suitable for EVT.

## Methods

### Study design

This study is based on data from the Oslo Acute Reperfusion Stroke Study (OSCAR), a prospective, observational study of consecutive stroke patients treated with EVT at Oslo University Hospital (OUH). OUH is a highly specialized regional university hospital and was responsible for the thrombectomy service to a population of 3.1 million inhabitants during the study inclusion period. Most patients were recruited from primary stroke centers (“drip and ship” model). In the period from January 1st, 2017, to December 31st 2021, a total of 956 patients accepted for EVT were included in the OSCAR study, and 159 patients had pMeVO in the anterior circulation. Clinical information, imaging, and procedural data were analyzed to identify predictive factors for successful reperfusion and good functional clinical outcome. The patient information was obtained from the patient’s medical record. Risk factors for intracranial hemorrhage and clinical importance of embolization to new vessel territories were assessed.

### Inclusion and exclusion criteria

**Fig. 1:**
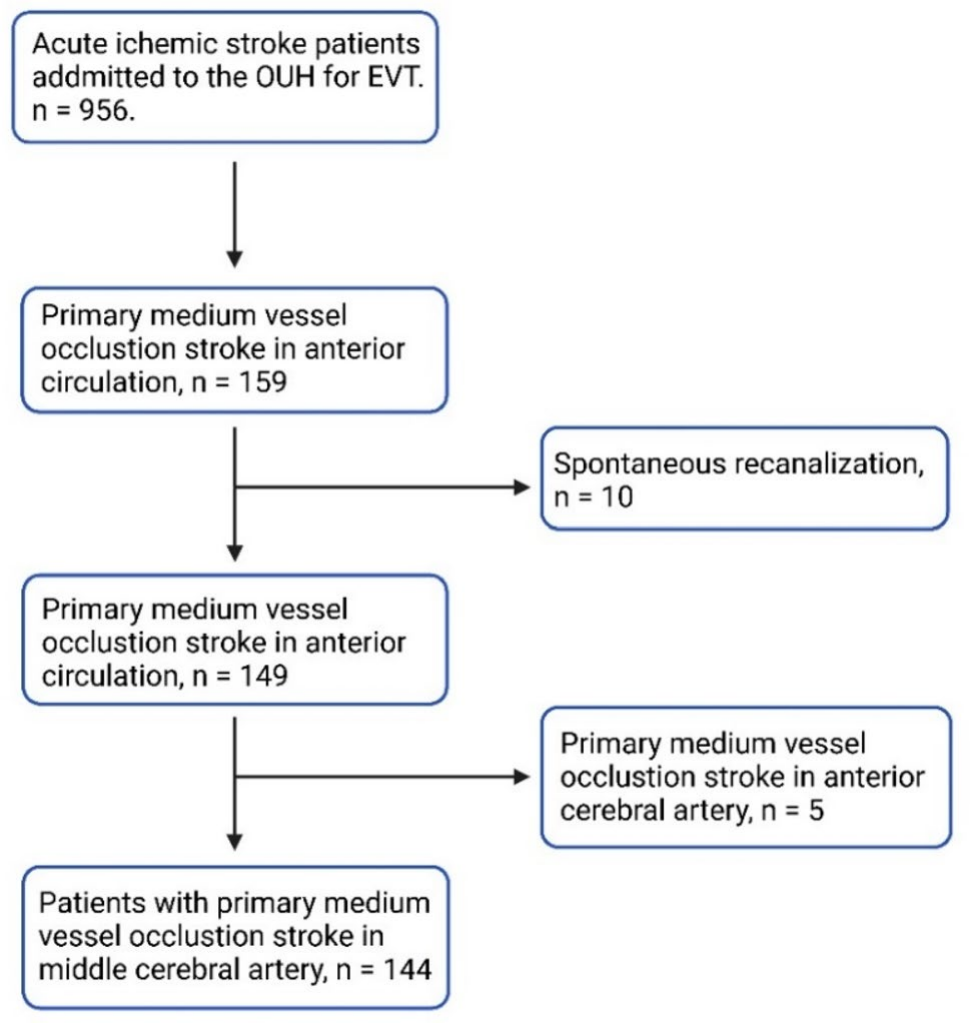
Study flowchart

A total of 154 patients were identified with pMeVO in the MCA territory. Ten patients were excluded due to partial or complete recanalization either spontaneously or due to thrombolysis with intravenous tissue plasminogen activator (IV-tPA) when examined with digital subtraction angiography (DSA). A total number of 144 patients with pMeVO in the MCA territory were included in the present study (see Fig. 1).

### Background clinical and imaging data

Data on patient demographics, pre-stroke functional level assessed by modified Rankin scale score (mRS), risk factors, comorbidity and antiplatelet or anticoagulation medication were registered (Table 1). Clinical parameters including National Institutes of Health Stroke Scale (NIHSS) score on admission and at discharge, and mRS at 90-day follow-up were prospectively registered. Patients with wake-up stroke or patients who were found with stroke symptoms were grouped together as unknown ictus.

**Table 1:** Baseline characteristics in acute anterior circulation stroke patients with medium-vessel occlusions undergoing endovascular treatment

Background and procedural characteristics of patient group	
Age, years^a^	70 (34–93)
Sex, male	77 (53.5%)
On antiplatelet drugs	45 (31.2%)
On anticoagulation drugs	34 (20.8%)
Currently smoking	29 (20.1%)
Hypertension	80 (55.6%)
Atrial fibrillation	71 (49.3%)
Diabetes mellitus	28 (19.4%)
Heart failure	25 (17.4%)
Previous stroke or TIA	42 (29.2%)
Pre-stroke mRS ≤ 2	134 (93.1%)
Unknown ictus	47 (32.6%)
Time from known ictus to recanalization, min^a^	312 (100–1140)
Time from found with symptoms to recanalization to, min^a^	370 (142–964)
Time from procedure start to recanalization, min^a^	51 (14–184)
M2 occlusion	125 (86.8%)
M3 occlusion	19 (13.2%)
Median ASPECT score on last imaging	8 (3–10)
Median CT ASPECT score	10 (5–10)
Median MRI ASPECT score	7 (3–10)
ASPECT ≥ 8	85 (59%)
ASPECT ≥ 8 CT	42 (89.4% of the group with CT)
ASPECT ≥ 8 MRI	43 (44.8% of the group with MRI)
IV-tPA	74 (51.4%)
Conscious sedation during EVT	113 (78.5%)
General anesthesia during EVT	31 (21.5%)
Only distal aspiration	13 (9.0%)
0–1 passes with stent retriever	84 (58.3%)
≥ 1 passes with stent retriever	60 (41.7%)
mTICI score ≥ 2B	122 (84.7%)
mTICI score ≥ 2C	82 (56.9%)
Embolization to new artery	16 (11.1%)
Cone beam CT scan after procedure	81 (56.2%)
SAH on cone beam CT scan	28 (19.4%)
SAH after 24 h	27 (18.8%)
Petechiae after 24 h	42 (29.2%)
Hematoma after 24 h	11 (7.1%)
Symptomatic hemorrhage	2 (1.4%)
mRS ≤ 2 at 90-day follow-up	84 (61.8%)
mRS ≤ 1 at 90-day follow-up	65 (47.8%)
Death after 90 days	25 (17.4%)

*MeVO* Medium vessel occlusion stroke, *TIA* transient ischemic attack, *mRS* modified Rankin scale, *NIHSS* National Institute of Health Stroke Scale, *IV-tPA* intravenous tissue-type plasminogen activator, *EVT* endovascular treatment, *mTICI* modified thrombolysis in cerebral infarction, SAH subarachnoid hemorrhage.

^a^Values are expressed as mean

^b^Values are expressed as median

The initial imaging was mainly performed at the referring hospitals. Unenhanced CT and CT angiography with or without CT perfusion were utilized to identify candidates for EVT. Many patients with wake-up stroke or unknown ictus had in addition brain MRI with diffusion-weighted imaging (DWI). Eligible patients for IV-tPA received treatment before transfer to our hospital or at our institution for candidates directly admitted or in-house strokes. In cases with long interhospital transfer time, altered clinical presentation or uncertain treatment indication upon arrival, an additional MRI or CT scan was done before EVT. Good functional outcome was defined as mRS score of 0–2 after 90 days. Patients were grouped into good or poor clinical outcome.

### EVT procedure

The EVT procedure was performed either on a Philips NeuroSuite with the Allura FD20/15 X-ray system (Philips, Eindhoven) or on a Siemens ARTIS zee biplane angiography system (Siemens, Erlangen). Recanalization procedure was performed either in general anesthesia (GA) or under conscious sedation (CS). Cerebral digital subtraction angiography (DSA) and EVT was performed via femoral access. Balloon catheter or long sheet was placed in internal carotid artery. EVT of the intracranial occlusion was performed either by direct aspiration alone, with stent retriever combined with distal aspiration or by combining these methods. The interventional neuroradiologist on duty was responsible for the method of choice. A total of 8 patients with pre-stroke mRS > 2 were treated with EVT after a thorough assessment were done by the intervention team. In patients with increased mRS prior to the stroke, the pre-morbid quality of life, the presence of penumbra on imaging and other mitigating factors were included in the assessment.

### Follow-up imaging

During the inclusion period, a routine head CT scan in the angiography lab (Cone beam CT) immediately after completion of the EVT procedure was introduced. Eighty-one of the 144 patients had an early Cone beam CT. All except four patients underwent either MRI or non-contrast enhanced CT follow-up within 24 h after endovascular therapy. Follow-up MR was performed on four different Siemens 1.5 and 3T machines, with a standardized protocols including diffusion-weighted imaging (DWI), susceptibility weighted imaging (SWI) and fluid-attenuated inversion recovery (FLAIR) sequences.

### Imaging analysis

The last CT or MRI examination prior to performing thrombectomy was evaluated retrospectively with regard to ASPECTS by two experienced neuroradiologists by consensus. When evaluating DWI-ASPECT, only DWI lesions with diameter ≥ 10 mm, visible on at least two adjacent DWI slices in one region were counted. The mTICI score was rated retrospectively based on the final DSA angiogram by evaluating the degree of recanalization solely in the initially occluded vessel territory [3]. Scoring was done by two independent, experienced interventional neuroradiologists, blinded to clinical information and outcome. Disagreement was resolved by consensus. Successful reperfusion was defined as mTICI ≥ 2b. Excellent reperfusion was defined as mTICI ≥ 2c [12].

The Heidelberg bleeding classification (HBC) was used to score intracranial hemorrhage. The scoring was evaluated by one experienced interventional neuroradiologist blinded to clinical information and outcome. Worsening of NIHSS score with ≥ 4 units together with detection of intracranial hemorrhage was classified as symptomatic intracranial hemorrhage (sICH).

### Definition of pMeVO in the MCA territory

In this study, we have included patients with M2 and M3 occlusions. As there are different ways to distinguish between LVO and MeVO [13] the M2 segment in this study was defined as distal from the MCA bifurcation/trifurcation to the circular sulcus of the insula. The M3 segment was defined as distal from the circular sulcus of the insula to the external/superior surface of the Sylvian fissure. The diagnosis of pMeVO was made on the initial CTA examination.

### Statistical analyses

Statistical analyses were performed using IBM SPSS Statistics (Version 29). Graphs were made in GraphPad Prism version 9.0.0 for Windows, GraphPad Software (San Diego, California USA, www.graphpad.com). Continuous variables were expressed as mean with minimum and maximum value or mean with standard deviation; SD. Categorical variables were expressed as number (percentage). Continuous variables were analyzed using binary logistic regression. Categorical variables were analyzed using Chi-square test. Values for time to recanalization exceeding three standard deviations from mean were considered extreme values and excluded from further analysis. For analysis with more than 2 variables, logistic regression was used. This type of analysis was performed for investigating the effect of thrombolysis, age, NIHSS, time from ictus to recanalization and number of passes on outcome after 3 months.

### Ethics

The OSCAR study was approved by the regional committee for medical and health research (REK no *2015/1844*). All participants provided written informed consent in accordance with the Declaration of Helsinki or consent were obtained from their legal authorized representative.

## Results

### Patient baseline and procedural characteristics

A total of 144 patients with pMeVO in the MCA territory were included in the study. The mean age was 70 years and 27.8% of the patients were 80 years or older, and 46.7% were female. Hypertension and atrial fibrillation were the most frequent cardiovascular risk factors (Table 1). Median ASPECT score was 8, and 85 patients (59.0%) had ASPECT score 8 or higher. ASPECT score was lower in the 96 patients with MRI ASPECT (7) compared to the 47 patients with CT ASPECT (10). M2 occlusion was the most common artery occlusion (84.7%). The median NIHSS score upon arrival was 10, and 5 at discharge. Thrombectomy was performed under either conscious sedation (78.5%) or general anesthesia (21.5%). Baseline and procedural characteristics are displayed in Tables 1 and 2.

**Table 2:** Relation between the number of passes during endovascular therapy and clinical and radiological variables in acute anterior circulation stroke patients with medium-vessel occlusions

Effect of number of passes			
	0–1 passes, *n* = 84	> 1 passes, *n* = 60	*p*-value
Time to recanalization, min^a^	320	348	0.260^&^
Time to recanalization (known ictus), min^a^	312	313	0.974^&^
Time from procedure start to recanalization, min^a^	39	69	< 0.001^&^
Median ASPECT score on last imaging	8 (5–10)	8 (3–10)	0.252^&^
Median CT ASPECT score	10 (5–10)	10 (6–10)	0.581^&^
Median MRI ASPECT score	7 (5–10)	7 (3–10)	0.535^&^
NIHSS on admission	10	10	0.375^&^
NIHSS at discharge	4	8.5	0.002^&^
IV-tPA	41 (48.8%)	33 (55.0%)	0.464*
Conscious sedation during EVT	68 (81%)	46 (76.7%)	0.728*
General anesthesia during EVT	16 (19%)	14 (23.3)	
Embolization new artery	8 (9.5%)	8 (13.5%)	0.473*
mTICI score ≥ 2B	78 (92.9%)	44 (73.3%)	0.001*
mTICI score ≥ 2C	59 (70.2%)	23 (38.3%)	< 0.001*
SAH on Cone Beam CT scan	5 (6%)	23 (38.3%)	< 0.001*
SAH after 24 h	6 (7.1%)	21 (35%)	< 0.001*
Petechiae after 24 h	20 (23.8%)	22 (36.7%)	0.085*
Haematoma after 24 h	7 (8.3%)	4 (6.7%)	0.722*
mRS ≤ 2 at 90-day follow-up	56 (66.7%)	28 (46.7%)	0.010*
mRS ≤ 1 at 90-day follow-up	44 (55.7%)	21 (36.8%)	0.030*
Dead after 90 days	12 (15.2%)	13 (22.8%)	0.258*

*NIHSS* National Institute of Health Stroke Scale, *IV-tPA* intravenous tissue-type plasminogen activator, *EVT* endovascular treatment, *mTICI* modified thrombolysis in cerebral infarction, SAH subarachnoid hemorrhage.

*Analyzed with Chi-square test

^&^Analyzed with logistic regression

^a^Values are expressed as mean

^b^Values are expressed as median

Nighty-seven patients (67.4%) had known ictus, whereas 29 patients (20.1%) had wake-up stroke and 18 (12.5%) were found with symptoms without known ictus. In patients found with symptoms without known ictus or with wake-up stroke, time from discovered ictus to recanalization was registered. In 79 out of the 97 patients with known ictus, EVT was started within 6 h after stroke onset. Regarding good outcome (mRS ≤ 2), there was no significant difference between patients who started EVT within 6 h after stroke onset and patients with unknown ictus or who started EVT after 6 h.

**Fig. 2:**
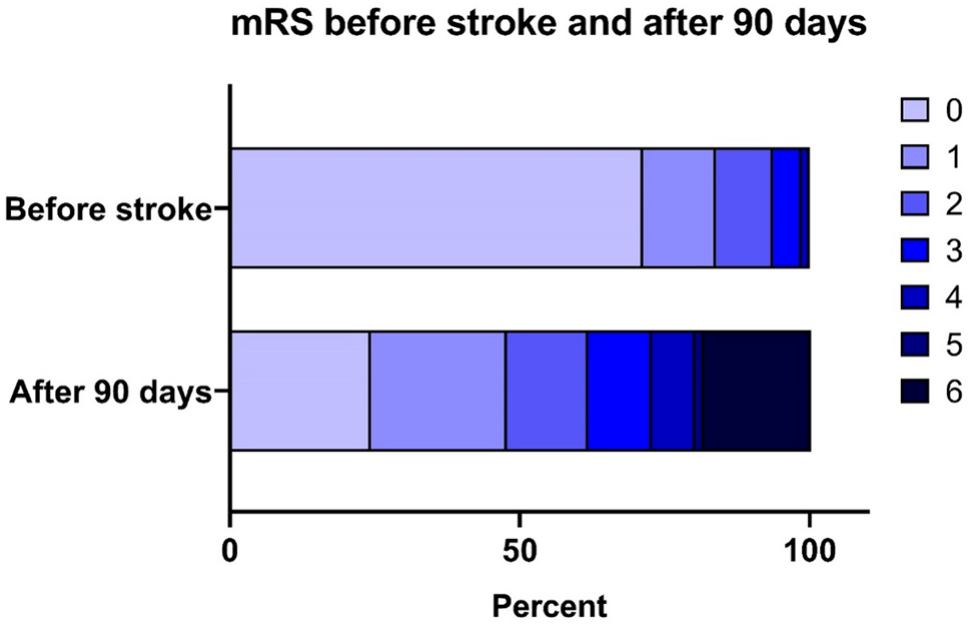
Modified Rankin scale (mRS) in the study population before stroke and 90 days after endovascular therapy

Distal aspiration EVT alone was done in 13 patients (9%) while stent retriever in combination with distal aspiration was done in 131 (91%). A successful recanalization (TICI score ≥ 2b) was achieved in 122 patients (84.7%). In 46.5% of patients, hemorrhage was detected 24 h after procedure. 84 patients (61.8%) had a favorable mRS outcome after 3 months (Fig. 2).

*Thrombolysis* Intravenous thrombolysis was administered in 51.4% of the patients. In patients not receiving IV-tPA, the majority had either unknown ictus, was beyond the time window for administration (4.5 h) or were on anticoagulation therapy. In three patients with ongoing cancer treatment, IV-tPA was withheld due to risk of hemorrhage. Two patients were in-house strokes after cardiac procedures and did not receive thrombolysis due to perioperative administration of anticoagulants/heparin. To investigate the effect of thrombolysis, we performed a logistic regression with the most known predictors for outcome including thrombolysis, age, NIHSS, time from ictus to recanalization and number of passes on outcome after 3 months. Age (*p* = 0.024), NIHSS arrival (< 0.001), number of passes with EVT (*p* = 0.006) was significantly associated with outcome, whereas IV-tPA (*p* = 0.629) and time (*p* = 0.220) were not.

### Technical factors

The number of passes is one of the main risk factors for poor outcome in LVO stroke [14–16]. Patients with one pass or distal aspiration alone obtained significant increased recanalization rate and better functional outcome. There were no significant differences in distribution of risk factors between patients with 0–1 passes with stent retriever and patients with > 1 passes. Patients with > 1 passes had significant longer procedure time, but time from ictus to recanalization was comparable between the groups. Two or more passes with stent retriever were also associated with decreased TICI score and increased NIHSS at discharge (Table 2). We found that passes > 1 with stent retriever significantly increased the risk of SAH after the procedure, both on the cone beam CT and after 24 h (Table 2).

### Embolization to new artery

In our study, 16 patients had embolization to a new artery during EVT (Table 1). No significant differences in background or procedural factors were observed between these patients and rest of the study participants nor were there any difference in mortality after 3 months.

### Symptomatic hemorrhage

Many patients experienced different type and degrees of intracranial hemorrhage, though SAH or petechiae were most common (Table 1). Eleven patients (7.1%) experienced haematoma (HBC 1C or 2) within 24 h while only two patients had sICH (1.38%). Patients with EVT procedure time over 60 min had increased risk of intracranial hemorrhage after 24 h (*p* 0.030).

### Gender perspectives

Comparing gender in this study, median age was 72 years in males and 73 years in females. Twenty-six of males and 29.9% of females were 80 years or older (Table 1). Among risk factors for cardiovascular disease, smoking, previous stroke and arterial fibrillation were more common in men, whereas diabetes was slightly more common in women. The pre-stroke mRS score was relatively equal in both sexes. There were no significant differences between the groups regarding known symptom onset, administration of IV thrombolysis or NIHSS on admission. 35 women (52.2%) and 50 men (64.9%) had ASPECT score 8 or higher on the last scan before EVT. In women, CS (82.6%) was used more frequently than in men (71.2%).

Time is one of the most important factors for clinical outcome in stroke. The mean time from onset to recanalization in men (300 min) was significantly shorter than in women (368.2 min) (*p* 0.01). In 77.9% of men, EVT was completed within 6 h, whereas only 59.7% of females completed the procedure in the same time window. There were no differences between the groups regarding percentage of unknown ictus (see Table 3).

**Table 3:** Clinical and procedural characteristics of male and female acute anterior circulation stroke patients with medium-vessel occlusions undergoing endovascular therapy

Clinical and procedural characteristics of men and women			
	Men, *n* = 77	Women, *n* = 67	*p*-value
On antiplatelet drugs	22 (28.6%)	24 (35.8%)	0.352*
On anticoagulation drugs	20 (26.0%)	14 (20.9%)	0.474*
Currently smoking	18 (23.4%)	11 (16.4%)	0.404*
Hypertension	43 (55.8%)	37 (55.2%)	0.940*
Atrial fibrillation	41 (53.2%)	30 (44.8%)	0.311*
Diabetes mellitus	14 (18.2%)	14 (20.9%)	0.682*
Heart failure	16 (21.2%)	9 (13.6%)	0.274*
Pre-stroke mRS ≤ 2	72 (93.5%)	62 (92.5%)	0.589*
Unknown ictus	24 (31.2%)	23 (34.3%)	0.687*
Time to recanalization, min^a^	300	368	0.010^&^
Time to recanalization known ictus, min^a^	284	347	0.033^&^
Time from procedure start to recanalization, min^a^	52	50	0.997^&^
Median ASPECT score on last imaging	8 (3–10)	8 (5–10)	0.341^&^
Median CT ASPECT score	10 (6–10)	10 (5–10)	0.085^&^
Median MRI ASPECT score	7 (3–10)	7 (5–10)	0.703^&^
NIHSS on admission^b^	10	10	0.317^&^
NIHSS at discharge^b^	5	5	0.791^&^
IV-tPA	37 (48.1%)	37 (55.2%)	0.390*
Conscious sedation during EVT	56 (72.7%)	58 (86.6%)	0.124*
General anesthesia during EVT	21 (27.3%)	9 (13.4%)	
0–1 passes with stent retriever	42 (54.5%)	42 (62.7%)	0.323*
≥ 1 passes with stent retriever	35 (45.5%)	25 (37.3%)	
mTICI score ≥ 2B	64 (83.1%)	58 (86.6%)	0.566*
mTICI score ≥ 2C	42 (45.5%)	40 (59.7%)	0.533*
Embolization to new artery	13.0%	9.0%	0.443*
SAH on cone beam CT scan	11 (14.3%)	17 (25.4%)	0.187*
SAH 24 h	13 (16.9%)	14 (20.9%)	0.529*
Petechiae 24 h	28 (36.4%)	14 (20.9%)	0.420*
Haematoma 24 h	6 (7.8%)	5 (7.5%)	0.946*
mRS ≤ 2 at 90-day follow-up	48 (62.3%)	36 (53.7%)	0.552*
mRS ≤ 1 at 90-day follow-up	34 (45.3%)	31 (50.8%)	0.524*
Dead after 90 days	13 (16.9%)	12 (17.9%)	0.726*

*NIHSS* National Institute of Health Stroke Scale, *IV-tPA* intravenous tissue-type plasminogen activator, *EVT* endovascular treatment, *mTICI* modified thrombolysis in cerebral infarction, SAH subarachnoid hemorrhage, *mRS* modified Rankin scale.

^a^Values are expressed as mean

^b^Values are expressed as median

*Analyzed with Chi-square test

^&^Analyzed with logistic regression

Although women had longer onset to recanalization time, no gender difference in TICI score was found. Incidence of hemorrhage was significantly higher in men (51.9%) than women (40.3%), though most of which were asymptomatic (HBC 1A and 1B). MRS was comparable in both male and female patients.

### MeVO in the elderly patients

**Fig. 3:**
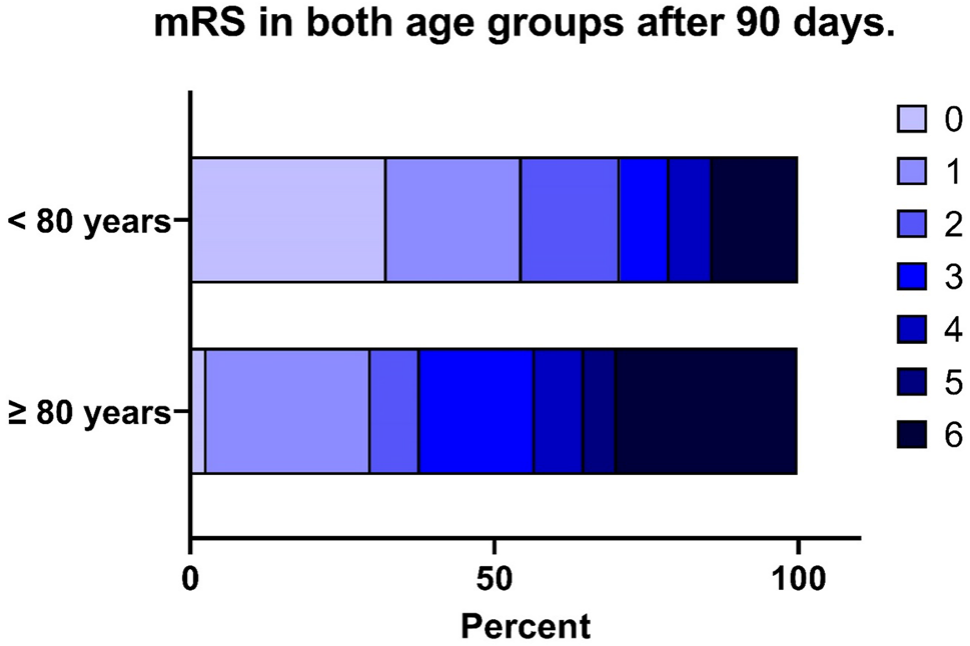
Modified Rankin scale (mRS) in patients under 80 years or 80 years and older 90 days after endovascular therapy

Dividing the study population into < 80 years and ≥ 80 years of age, we found no significant differences regarding administration of IV-TPA, NIHSS upon arrival or time to recanalization (Table 4). Technical factors, mTICI score and intracranial hemorrhage were also comparable. Patients above 80 years, however, had higher NIHSS at discharge and had significantly increased risk of both poor functional outcome and death after 3 months (Fig. 3).

**Table 4:** Clinical and procedural information in patients under or above 80 years undergoing endovascular therapy for medium-vessel occlusions

Clinical and procedural information in patients under or above 80 years			
	< 80 years, *n* = 104	80 years or older, *n* = 40	*p*-value
Pre-stroke mRS ≤ 2	99 (95.2%)	35 (87.5%)	0.057*
Unknown ictus	32 (30.8%)	15 (37.5%)	0.440*
IV-tPA	53 (51.0%)	21 (52.5%)	0.869*
Time to recanalization, min^a^	333	328	0.841^&^
Time to recanalization (known ictus), min^a^	312	315	0.933^&^
Time from procedure start to recanalization, min^a^	51	53	0.697^&^
Median ASPECT score on last imaging	8 (4–10)	8 (3–10)	0.688^&^
Median CT ASPECT score	10 (6–10)	10 (5–10)	0.737^&^
Median MRI ASPECT score	7 (4–10)	7.5 (3–10)	0.485^&^
NIHSS on arrival^a^	10	10,5	0.269^&^
NIHSS at discharge	4.5	7	0.013^&^
Conscious sedation during EVT	84 (80.8%)	30 (75.0%)	0.297*
General anesthesia during EVT	17 (19.2%)	10 (25.0%)	
0–1 passes with stent retriever	62 (59.6%)	22 (55.0%)	0.615*
> 1 passes with stent retriever	42 (40.4%)	18 (45.0%)	
Embolization new artery	11 (10.6%)	5 (12.5%)	0.742*
mTICI score ≥ 2B	90 (86.5%)	32 (80.0%)	0.329*
mTICI score ≥ 2C	64 (61.5%)	18 (45.0%)	0.073*
SAH on cone beam CT scan	18 (17.3%)	10 (25%)	0.875*
SAH after 24 h	10 (19.2%)	7 (17.5%)	0.803*
Petechiae after 24 h	32 (30.8%)	10 (25%)	0.484*
Haematoma after 24 h	9 (8.7%)	2 (5.0%)	0.456*
mRS ≤ 2 at 90-day follow-up	70 (67.3%)	14 (35.0%)	< 0.001*
mRS ≤ 1 at 90-day follow-up	54 (54.4%)	11 (29.7%)	0.010*
Dead after 90 days	14 (13.5%)	11 (27.5%)	0.037*

*mRS* modified Rankin scale, *NIHSS* National Institute of Health Stroke Scale, *IV-tPA* intravenous tissue-type plasminogen activator, *EVT* endovascular treatment, *mTICI* modified thrombolysis in cerebral infarction, SAH subarachnoid hemorrhage

*Analyzed with Chi-square test

^&^Analyzed with logistic regression

^a^Values are expressed as mean

## Discussion

This study shows that acute ischemic stroke patients with MeVO in the MCA territory treated with either aspiration alone or a single pass with stent retriever achieved higher mTICI score and increased probability of good outcome compared to patients with > 1 pass. In addition, the risk for SAH was increased with > 1 pass. Embolization to new vessel territory, however, did not affect the 90-day outcome. On average, time from ictus to recanalization was 1 h longer in women than in men, though not influencing clinical outcome. Patients ≥ 80 years had worse 90-day outcome and increased mortality compared to younger patients.

These results are based on a prospective, observational study of consecutive stroke patients treated with EVT at our hospital and reflect the daily practice. Due to the organization of the thrombectomy service at the time, and coverage for a large geographical area, a very large proportion (98%) of the study population are “drip and ship”” patients. This is expressed in a relatively long time from ictus to recanalization which likely affect the functional outcome. In our material, we had a higher recanalization rate (mTICI ≥ 2B) in 84.7% compared to the HERMES study (59.2%) [8]. Despite this, the number of patients with good clinical outcome (mRS ≤ 2) after 90 days was almost equal (61.8% vs 58.2% in the HERMES study). Longer time from ictus to recanalization and higher median age in our participants might explain this.

We found no significant difference between patients who started EVT within 6 h after stroke onset and patients with unknown ictus or who started EVT after 6 h. This finding might be explained by a more restrictive selection of candidates for thrombectomy in the extended time window, as well as for patients with unknown ictus. First pass effect (FPE), defined as successful revascularization after a single stent-retriever pass (mTICI ≥ 2B), has been shown to be linked to better clinical outcome compared with non-FPE in patients with LVO [14–16]. This study showed the same impact of FPE in patients with pMeVO with a significantly increased probability of both mRS ≤ 1 and mRS ≤ 2.

SAH following mechanical thrombectomy has been shown to be associated with distally located vessel occlusions and a higher number of thrombectomy device passes required to achieve reperfusion [17]. In our study, 19% had SAH after 24 h. The use of MRI including SWI sequence in the 24-h follow-up as well as the utilization of cone beam CT may explain the relatively high rate of SAH and other hemorrhagic complications. We found a highly significant correlation between the occurrence of SAH and number of passes with stent retriever. However, there was no significant correlation between SAH and 90-day outcome.

Review and meta-analysis of randomized controlled trials supports that in ischemic stroke patients treated with EVT, general anesthesia (GA) is associated with higher recanalization rates and improved functional recovery at 3 months compared with non-GA techniques [18]. It is likely that patients with peripheral occlusions benefit in utilizing GA, reducing the challenges related to navigation in small peripheral arteries [10]. In the last 3 years, there is a clear tendency in our institution towards increased use of GA during thrombectomy procedures, especially for peripheral occlusions. This tendency is to a small extent expressed in this study where only 21.5% of the procedures were performed under GA. Only 1 out 16 patients with embolization to new vessel territory had GA during the procedure, and our analysis showed no significant correlation between type of anesthesia and likelihood for embolization to new vessel territory.

No difference in clinical outcome was found in patients with embolization to a new artery in this study. This was unexpected, as one would suppose that embolization would lead to new infarctions and worse neurological function. A possible explanation could be that embolization occurred in smaller branches supplying smaller vascular territories. In addition, there are insufficient data to determine the extent to which the peripheral embolization led to new infarcts.

Previous studies focusing on sex differences in LVO stroke patients treated with EVT has not found significant differences regarding time from ictus until recanalization [19]. In our study, the mean time from ictus to recanalization in women was 1 h more than in men, however, without significant influence on clinical outcome. Known risk factors were not differently distributed in men and women. There were no obvious differences in number of wake-up strokes, age or other parameters that might explain this delay in treatment. Recent meta-analysis [20, 21] describes that men and women may have different spectrums of stroke symptoms, with women presenting non-focal symptoms to a greater extent than men. This may be part of the explanation for the difference, but we do not have detailed data to verify this. If a higher proportion of the women lived alone when they had the stroke, this could explain the difference in time to recanalization; however, there are no data available to substantiate this hypothesis.

It is even more difficult to explain why delayed time to recanalization in women did not lead to a reduced functional outcome. However, a higher proportion of women had thrombectomy performed by aspiration alone or with only one pass with stent retriever and they obtained a slightly higher mTICI score than men, which potentially may have contributed to better outcome and thus compensated for longer time to recanalization.

In patients aged 80 years and older, only 35% achieved good clinical outcome at 3 months compared to 67.3% in patients younger than 80 years. There was no significant difference in time to recanalization between the two groups that could explain this difference in outcome. Data from the Dutch MR CLEAN Registry with 380 patients aged ≥ 80 demonstrated worse functional outcome and higher mortality after EVT than in younger patients. Clinical frailty has been demonstrated to be associated with 28-day mortality after ischemic stroke and with poor improvement in NIHSS following stroke thrombolysis [22]. Similar findings have been reported in other studies focusing on the significance of frailty for functional outcome after EVT in elderly patients [23]. Pooled analyses of individual patient data from randomized trials and registry studies, however, support a positive benefit–risk profile of IV-tPA for acute ischemic stroke, among patients aged > 80 years, when administered according to other European regulatory criteria [24].

A meta-analysis of the randomized controlled EVT studies included 198 patients aged 80 years and older showed a favorable effect of EVT on modified Rankin scale (mRS) [1]. However, there is little documentation on the beneficial effect of EVT in elderly patients with pMeVO. This raises the question whether elderly patients with pMeVO should have stricter selection criteria for EVT than younger patients. According to our findings elderly patients should be carefully selected, as multimorbidity, increased pre-mRS and age, indicate reduced benefit. Screening for frailty might be helpful in the selection process.

## Conclusion

TICI score and number of passes with stent retriever were the main technical factors predicting outcome (mRS) after 3 months in pMeVO patients. Good clinical outcome was about twice as often in patients under 80 years of age compared to patients over 80 years of age. Women with MeVO strokes had significant longer time from ictus to recanalization; however, this did not affect the clinical outcome in women in the present study.

## Data Availability

Raw data for dataset D1 are not publicly available to preserve individuals’ privacy under the European General Data Protection Regulation. The data that support the findings of this study are available, but restrictions apply to the availability of these data, which were used under license for the current study, and so are not publicly available. Data are however available from the authors upon reasonable request, for details please contact Anne Hege Aamodt (a.h.aamodt@medisin.uio.no).
